# Serum markers of CYFRA 21-1 and C-reactive proteins in oral squamous cell carcinoma

**DOI:** 10.1186/s12957-015-0656-9

**Published:** 2015-08-21

**Authors:** Yin-Ping Hsu, Chia-Hsun Hsieh, Hui-Tzu Chien, Chi-Hsiung Lai, Chung-Kan Tsao, Chun-Ta Liao, Chung-Jan Kang, Hung-Ming Wang, Joseph Tung-Chieh Chang, Shiang-Fu Huang

**Affiliations:** Department of Otolaryngology-Head and Neck Surgery, Chang Gung Memorial Hospital, Chang Gung University, No. 5, Fu-Shin Street, Kwei-Shan, Linkou, Taoyuan, 333 Taiwan, Republic of China; Department of Medical Oncology, Chang Gung Memorial Hospital, Chang Gung University, Taoyuan, Taiwan, Republic of China; Department of Public Health, Chang Gung University, Taoyuan, Taiwan, Republic of China; Department of Plastic and Reconstructive Surgery, Chang Gung Memorial Hospital Linkou Medical Center, Chang Gung University, Taoyuan, Taiwan, Republic of China; Department of Radiation Oncology, Chang Gung Memorial Hospital, Chang Gung University, Taoyuan, Taiwan, Republic of China

## Abstract

**Background:**

CYFRA 21-1 (cytokeratin 19 fragment) and C-reactive proteins (CRP) were separately reported to be associated with prognosis of head and neck squamous cell carcinoma. The combined roles of CYFRA 21-1 and CRP levels were rarely investigated in oral squamous cell carcinoma (OSCC). The purpose of the present study was to analyze the relationship between preoperative levels of both CYFRA 21-1 and CRP, with clinicopathological factors and prognosis in OSCC patients.

**Methods:**

A retrospective study was performed on 130 OSCC patients between December 2010 and June 2013. Their serum CYFRA 21-1 and CRP levels were measured preoperatively.

**Results:**

CYFRA 21-1 level of ≥3.3 ng/mL and CRP level of ≥5.0 mg/L were significantly associated with pathological tumor status (*P* < 0.001), tumor depth (>10 vs. ≤10 mm, *P* = 0.001), bone invasion (*P* = 0.001), skin invasion (*P* = 0.006), pathologic nodal metastasis (*P* = 0.012), and disease-free survival (*P* = 0.009). Higher CYPFRA 21-1 and CRP levels were also associated with higher risks of distant metastasis (log-rank test, *P* = 0.013, (HR [95 % CI]) 1.692 [1.097–2.414]).

**Conclusions:**

Preoperative CYFRA 21-1 and CRP levels are probable candidates as biomarkers for risk stratification in OSCC.

## Background

Oral cavity cancer is one of the commonest cancers in the world but shows wide geographical variation due to habitual consumption of cigarette, alcohol, and areca quid (AQ). The incidence of oral cavity cancer ranks fifth among the types of cancer in Taiwan [1]. Search for significant biomarkers predicting tumor behaviors and patients’ prognosis may help clinicians choose appropriate treatment for those patients [2–7]. Newer markers that can help us better and more precisely predict patients’ prognosis are needed clinically.

In oral squamous cell carcinoma (OSCC), to early detect tumor, it usually links to the squamous cell component of cancer. Cytokeratins are structural proteins forming the subunits of epithelial intermediary filaments. In the literature, 20 different cytokeratin polypeptides have been identified. Cytokeratin 19 is expressed by normal and benign epithelial cells and various carcinomas, particularly the lung cancer. CYFRA 21-1 is the serum soluble fragment of cytokeratin 19 and was first described in the mid 1990s [8]. Increased concentration of CYFRA 21-1 was shown to be associated with poorer prognosis in patients with lung cancer [9, 10]. The measurement of CYFRA 21-1 in patients with squamous cell carcinoma of the head and neck (HNSCC) is also an established tumor marker and prognosticator [8, 11–17]. CYFRA 21-1 serum levels are significantly higher in patients with HNSCC compared to a healthy or control group [8]. Sawant et al. [18] reported a sensitivity of 84 % and a specificity of 93 % of CYFRA 21-1 in patients with oropharyngeal cancer. They found the serum marker reduced significantly after surgical therapy of the primary tumor.

A second potentially significant marker is the acute phase protein CRP, which has also been shown to correlate with survival in human cancers [3, 19–22]. We previously demonstrated that C-reactive protein (CRP) elevation in OSCC is associated with poor survival and tumor invasiveness [2, 3]. Elevated CRP could be a marker for chronic inflammation in the tumor microenvironment, with chronic inflammation itself also acts as a stimulus for angiogenesis, cell proliferation, and tumorigenesis [23–25]. However, the relationship between CYFRA 21-1 and CRP, and their potential combined value as prognostic markers of survival, has not been previously explored in OSCC. In this study, we retrospectively analyzed 130 OSCC patients who were primarily treated with radical excision in our institution. The aim of this study was to evaluate the importance of CYFRA 21-1 and CRP as tumor markers in patients with OSCC at the time of initial diagnosis in correlation with tumor size, histologic grading, and lymph node metastasis.

## Methods

### Patients and staging workup

We retrospectively reviewed 130 consecutive OSCC patients who had undergone primary radical surgery and were subsequently followed at Chang Gung Memorial Hospital from December 2010 to June 2013. The serum samples were obtained prior to surgery. All patients underwent radical surgery with curative intent. The follow-up for each patient began at the time of cancer treatment and ended at the time of death or last time clinic follow-up, whichever came first.

The patients in this series underwent an extensive preoperative survey, which included a detailed medical history and a complete physical examination, and computed tomography (CT) or magnetic resonance imaging (MRI) scans of the head and neck. Abdominal sonography and bone scan or positron emission tomography (PET) were also included in preoperative tumor survey. The guidelines of the 2010 American Joint Committee on Cancer (AJCC) (tumor-node-metastasis (TNM) classification) were employed for clinical staging [26]. Patients who were initially diagnosed of a distant metastasis were excluded from the analysis.

### Treatment

One hundred twenty-seven participants underwent a wide excision of the primary tumors with 1-cm safe margins (both peripheral and deep margins), which were cryosectioned to ensure that the margin was free from the tumor tissue. Supraomohyoid or modified radical neck dissection was performed according to patients’ clinical nodal status. All histological parameters including the depth of infiltration, bone, skin invasion, lymph node extracapsular spread (ECS), and grade of differentiation were recorded.

Postoperative radiotherapy (RT) was performed in patients who presented a stage pT4 tumor, pathologically positive lymph nodes, or pathologically close margins (≤4 mm). Concomitant chemoradiotherapy (CCRT) with cisplatin-based agents was administered to patients with ECS or pathological multiple lymph node metastases [27, 28]. Three patients received CCRT first, and received salvage radical surgery due to persistence of disease.

### Follow-up

All of the patients had a checkup every month during the first 6 months after treatment, every 2 months during the following 6 months, every 3 months during the second year, and every 6 months thereafter. All the patients were subjected to a hemogram, blood chemistry, chest X-ray, and CT scan or MRI in the first 3 and 6 months and then annually afterward. Patients with abnormal clinical symptoms/signs or laboratory data during follow-up would receive a bone scan and liver ultrasound.

### Measurement of CYFRA 21-1

Cytokeratin 19 fragments were detected by the monoclonal antibodies KS 19.1 and BM 19.21; these antibodies are specific for two different epitopes of cytokeratin 19 [29]. The measurement of CYFRA 21-1 was completed in electrochemiluminescent immunoassay (ECLIA) using the CYFRA 21-1 reagent kit. The CYFRA 21-1 concentration of each sample was automatically calculated in a Roche Analytics E170 immunology analyzer. The calculated concentration of CYFRA 21-1 was expressed in ng/mL, and the cut-off level of 3.3 ng/mL was used according to the manufacturer’s instructions (Roche Diagnostics, Mannheim, Germany) [30]. CYFRA 21-1 serum levels were determined for each patient at the time of initial diagnosis.

### Measurement of CRP

Preoperative serum CRP levels were checked at the time of tissue diagnosis before any medical intervention or antibiotic treatment to minimize intra-individual differences. A fresh blood sample was collected and sent to the laboratory for testing. Serum CRP levels were detected using an auto-analyzer (Hitachi 7600-210, Hitachi Medico, Tokyo). The cut-off point for serum CRP was set at 5.0 mg/L, which is internationally adopted for inflammation [3, 5, 31].

### Ethics

The study was approved by the Institutional Review Board (103-3590B) of Chang Gung Memorial Hospital, Linkou, Taiwan, ROC.

## Results

### Patient characteristics

Table 1 shows the clinicopathological characteristics of the 130 OSCC patients (114 males and 16 females). The tongue (*N* = 58, 44.6 %) and the buccal mucosa (*N* = 44, 33.8 %) were the most common primary tumor sites. The tumor stage distribution was 28 (21.5 %) in stage I, 17 (13.1 %) in stage II, 23 (17.7 %) in stage III, and 62 (47.7 %) in stage IV. The median follow-up period was 19.0 months. All patients received radical surgeries, and the adjuvant therapies were listed in Table 1. All patients were followed in clinic at least 6 months after treatment.

**Table 1:** Characteristics of the 130 oral cavity squamous cell carcinoma patients

Characteristic	No. of patients (%)
Age (years)	
Mean	52.30
Range	29.0–84.0
Gender	
Male	114 (87.7)
Female	16 (12.3)
Site of primary tumor	
Tongue	58 (44.6)
Mouth floor	3 (2.3)
Lip	5 (3.8)
Buccal mucosa	44 (33.8)
Alveolar ridge	13 (10.0)
Hard palate	0 (0)
Retromolar trigone	7 (5.4)
Pathologic tumor status	
T1	36 (27.7)
T2	38 (29.2)
T3	13 (10.0)
T4a	30 (23.1)
T4b	13 (10.0)
Pathologic N stage	
N0	68 (52.3)
N1	25 (19.2)
N2b	30 (23.1)
N2c	7 (5.4)
Pathologic stage	
Stage I	28 (21.5)
Stage II	17 (13.1)
Stage III	23 (17.7)
Stage IV	62 (47.7)
Treatment	
Surgery alone	57 (43.8)
Surgery with adjuvant radiation therapy	12 (9.2)
Surgery with adjuvant chemoradiation therapy	58 (44.6)
Chemoradiation therapy plus surgery	3 (2.3)

### CRP levels, clinicopathological variables, and prognosis

Elevated CRP levels (CRP ≥5.0 mg/L) were found to be associated with the skin invasion (*P* = 0.009), bone invasion (*P* < 0.001), tumor depth (≥10 vs. <10 mm, *P* < 0.001), pathological tumor status (*P* < 0.001), and pathologic nodal metastasis (*P* = 0.049) (Table 2). Higher CRP level was also found to be related with lymph node metastasis with ECS (*P* = 0.014).

**Table 2:** The associations between preoperative CRP, CYFRA 21-1, and clinicopathologic parameters (*N* = 130)

	CRP			CYFRA 21-1		
	Negative (*n* (%))	Positive (*n* (%))	*P* value	Negative (*n* (%))	Positive (n (%))	*P* value
Pathologic tumor status						
Early^a^ (*n* = 74)	64 (86.5)	10 (13.5)	*<0.001* ^e^	66 (89.2)	8 (10.8)	0.308
Advanced^b^ (*n* = 56)	29 (51.8)	27 (48.2)		46 (82.1)	10 (17.9)	
Pathologic N stage						
N0 (*n* = 68)	54 (79.4)	14 (20.6)	*0.049*	59 (86.8)	9 (13.2)	*0.024*
N1 (*n* = 25)	18 (72.0)	7 (28.0)		25 (100.0)	0 (0.0)	
N2 (*n* = 37)	21 (56.8)	16 (43.2)		28 (75.7)	9 (24.3)	
Nodal status						
(−) metastasis, (−) ECS (*n* = 68)	54 (79.4)	14 (20.6)	*0.014*	59 (86.8)	9 (13.2)	*0.039*
(+) metastasis, (−) ECS (*n* = 23)	18 (78.3)	5 (21.7)		23 (100.0)	0 (0.0)	
(+) metastasis, (+) ECS (*n* = 39)	21 (53.8)	18 (46.2)		30 (76.9)	9 (23.1)	
Differentiation						
Well (*n* = 32)	21 (65.6)	11 (34.4)	0.304	29 (90.6)	3 (9.4)	0.689
Moderate (*n* = 80)	61 (76.2)	19 (23.8)		68 (85.0)	12 (15.0)	
Poor (*n* = 18)	11 (61.1)	7 (38.9)		15 (83.3)	3 (16.7)	
Tumor stage						
Early^c^ (*n* = 45)	40 (88.9)	5 (11.1)	*0.002* ^e^	38 (84.4)	7 (15.6)	0.791^e^
Advanced^d^ (*n* = 85)	53 (62.4)	32 (37.6)		74 (87.1)	11 (12.9)	
Skin invasion						
No (*n* = 117)	88 (75.2)	29 (24.8)	*0.009* ^e^	102 (87.2)	15 (12.8)	0.389^e^
Yes (*n* = 13)	5 (38.5)	8 (61.5)		10 (76.9)	3 (23.1)	
Bone invasion						
No (*n* = 105)	83 (79.0)	22 (21.0)	*<0.001* ^e^	91 (86.7)	14 (13.3)	0.750^e^
Yes (*n* = 25)	10 (40.0)	15 (60.0)		21 (84.0)	4 (16.0)	
Tumor depth ≥10 mm						
No (*n* = 60)	53 (88.3)	7 (11.7)	*<0.001* ^e^	54 (90.0)	6 (10.0)	0.311^e^
Yes (*n* = 70)	40 (57.1)	30 (42.9)		58 (82.9)	12 (17.1)	

*ECS* extracapsular spread

^a^T1–T2

^b^T3–T4

^c^Stage I–II

^d^Stage III–IV

^e^Fisher’s exact test

**Fig. 1:**
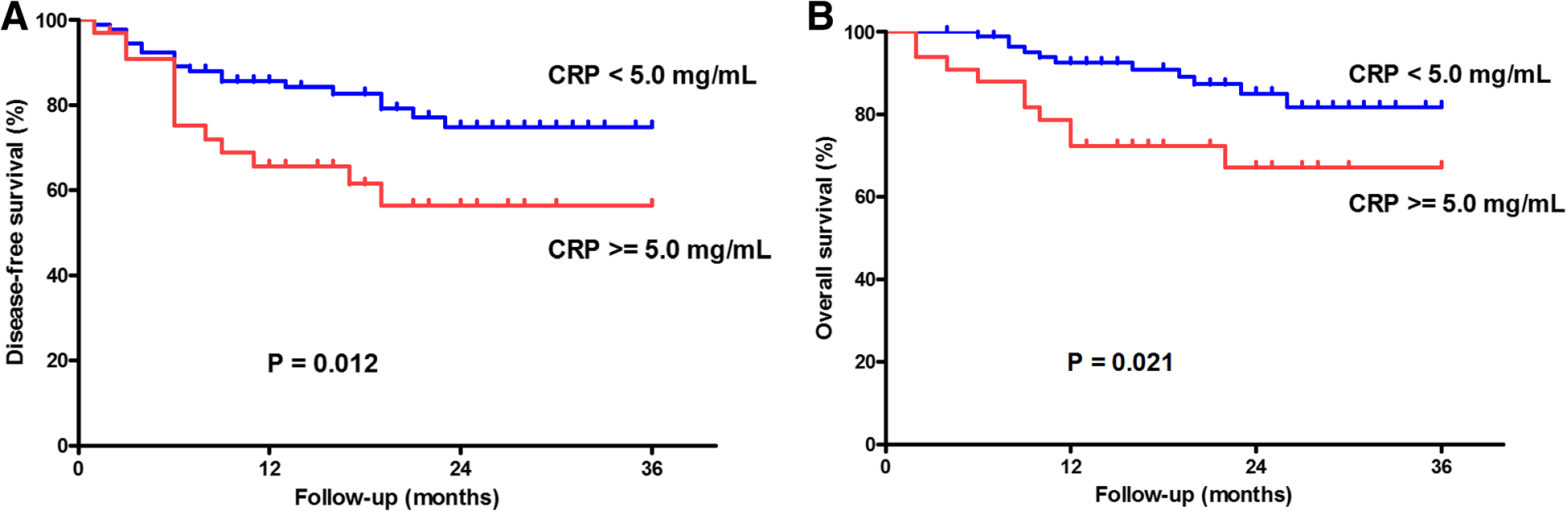
Survival curves in 130 OSCC patients related to the preoperative CRP level. **a** The lower CRP group (<5.0 mg/L) showed significantly better DFS compared to the higher CRP level group (≥5.0 mg/L) (*P* = 0.012). **b** The lower CRP group showed significantly better OS compared to the higher CRP level group (*P* = 0.021)

When comparing the survival differences between the higher CRP group (CRP ≥5.0 mg/L) and lower CRP group (CRP <5.0 mg/L), the disease-free survival (DFS) was significantly better in the latter than that of the former group (log-rank test, *P* < 0.012, Fig. 1a). In addition, in the higher CRP group (CRP ≥5.0 mg/L), the overall survival (OS) was also remarkably worse than that in the lower CRP group (CRP <5.0 mg/L) (log-rank test, *P* = 0.021, Fig. 1b).

### CYFRA 21-1 level and its relation with clinicopathological variables and prognosis

A higher CYFRA 21-1 level (CYFRA 21-1 ≥3.3 ng/mL) was statistically related with pathological nodal status (*P* = 0.024) and nodal ECS (*P* = 0.039). However, elevated CYFRA 21-1 level was not related with bone invasion, skin invasion, and tumor depth (Table 2).

**Fig. 2:**
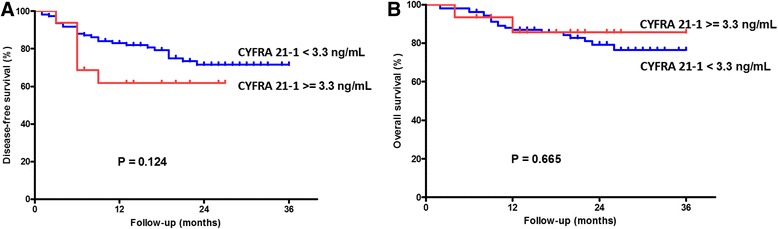
Survival curves in 130 OSCC patients related to the preoperative CYFRA 21-1. **a** The lower CYFRA 21-1 group (<3.3 ng/mL) showed better DFS compared to the higher CYFRA 21-1 level group (≥3.3 ng/mL) (*P* = 0.124). **b** The lower CYFRA 21-1 group showed similar OS compared to the higher CYFRA 21-1 level group (*P* = 0.665)

The DFS was insignificantly worse in the higher CYFRA 21-1 level group (CYFRA 21-1 ≥ .3 ng/mL) than that of the lower CYFRA 21-1 level group (CYFRA 21-1 <3.3 ng/mL) (log-rank test, *P* = 0.124, Fig. 2a). In addition, the OS was neither associated with the CYFRA 21-1 level (log-rank test, *P* = 0.665, Fig. 2b).

### Combined CRP and CYFRA 21-1 level and its relation with clinicopathological variables and prognosis

CRP level was related with CYFRA 21-1 level in linear correlation (*P* = 0.010). A higher CYFRA 21-1 level was accompanied by a higher serum CRP level. When the patients were divided into four groups according to the preoperative higher or lower CYFRA 21-1 and CRP levels, a close association was observed between a coexistence of higher CYFRA 21-1 (≥3.3 ng/mL) and higher CRP (≥5.0 mg/L) status and pathological tumor status (*χ*^2^ trend test *P* < 0.001), pathologic nodal metastasis (*χ*^2^ trend test *P* = 0.012), tumor stage (*P* = 0.009), bone invasion (*P* = 0.001), skin invasion (*P* = 0.006), and tumor depth (≥10 vs. <10 mm, *P* < 0.001) (Table 3).

**Table 3:** The associations between preoperative CRP, CYFRA 21-1, and clinicopathologic parameters (*N* = 130)

	CRP (−), CYFRA 21-1 (−)	CRP (−),CYFRA 21-1 (+)	CRP (+),CYFRA 21-1 (−)	CRP (+),CYFRA 21-1 (+)	
	(*n* (%))	(*n* (%))	(*n* (%))	(*n* (%))	*P* value
Tumor stage					
Early^a^ (*n* = 45)	33 (73.3)	7 (15.6)	5 (11.1)	0 (0.0)	*0.009* ^e^
Advanced^b^ (*n* = 85)	52 (61.2)	1 (1.2)	22 (25.9)	10 (11.8)	
Pathologic N stage					
N0 (*n* = 68)	47 (69.1)	7 (10.3)	12 (17.6)	2 (2.9)	*0.012* ^e^
N1 (*n* = 25)	18 (72.0)	0 (0.0)	7 (28.0)	0 (0.0)	
N2 (*n* = 37)	20 (54.1)	1 (2.7)	8 (21.6)	8 (21.6)	
Nodal status					
(−) metastasis, (−) ECS (*n* = 68)	47 (69.1)	7 (10.3)	12 (17.6)	2 (2.9)	*0.006* ^e^
(+) metastasis, (−) ECS (*n* = 23)	18 (78.3)	0 (0.0)	5 (21.7)	0 (0.0)	
(+) metastasis, (+) ECS (*n* = 39)	20 (51.3)	1 (2.6)	10 (25.6)	8 (20.5)	
Differentiation					
Well (*n* = 32)	20 (62.5)	1 (3.1)	9 (28.1)	2 (6.2)	0.734
Moderate (*n* = 80)	55 (68.8)	6 (7.5)	13 (16.2)	6 (7.5)	
Poor (*n* = 18)	10 (55.6)	1 (5.6)	5 (27.8)	2 (11.1)	
Pathologic tumor status					
Early^c^ (*n* = 74)	56 (75.7)	8 (10.8)	10 (13.5)	0 (0.0)	*<0.001* ^e^
Advanced^d^ (*n* = 56)	29 (51.8)	0 (0.0)	17 (30.4)	10 (17.9)	
Skin invasion					
No (*n* = 117)	80 (68.4)	8 (6.8)	22 (18.8)	7 (6.0)	*0.006* ^e^
Yes (*n* = 13)	5 (38.5)	0 (0.0)	5 (38.5)	3 (23.1)	
Bone invasion					
No (*n* = 105)	75 (71.4)	8 (7.6)	16 (15.2)	6 (5.7)	*0.001* ^e^
Yes (*n* = 25)	10 (40.0)	0 (0.0)	11 (44.0)	4 (16.0)	
Tumor depth ≥10 mm					
No (*n* = 60)	47 (78.3)	6 (10.0)	7 (11.7)	0 (0.0)	*<0.001* ^e^
Yes (*n* = 70)	38 (54.3)	2 (2.9)	20 (28.6)	10 (14.3)	

*CRP (−)* CRP level <5.0 mg/L, *CRP (+)* CRP level ≥5.0 mg/L, *CYFRA 21-1 (−)* CYFRA 21-1 <3.3 ng/mL, *CYFRA 21-1 (+)* CYFRA 21-1 ≥3.3 ng/mL, *ECS* extra-capsular spread

^a^Stage I–II

^b^Stage III–IV

^c^T1–T2

^d^T3–T4

^e^Fisher’s exact test

**Table 4:** Univariate log-rank test of prognostic covariates in 130 patients with oral cavity squamous cell carcinoma regarding disease-free and overall survival

	DFS, *P* value	OS, *P* value
	[HR (95 % CI)]	[HR (95 % CI)]
Age (years)		
<50	0.251	0.885
≥50	0.678 (0.349–1.316)	0.938 (0.395–2.228)
Sex		
Female	0.776	0.164
Male	1.163 (0.411–3.297)	0.490 (0.179–1.337)
Nodal status		
(−) metastasis, (−) ECS	*0.001*	*0.008*
(+) metastasis, (−) ECS	1.193 (0.384–3.705)	1.858 (0.463–7.447)
(+) metastasis, (+) ECS	3.586 (1.733–7.420)	4.618 (1.729–12.338)
Differentiation		
Well/moderate	*0.027*	*0.041*
Poor	2.437 (1.105–5.372)	2.868 (1.043–7.886)
Pathologic tumor status		
Early^a^	*0.023*	*0.096*
Advanced^b^	2.172 (1.111–4.247)	2.084 (0.877–4.949)
CRP		
<5 mg/mL	*0.016*	*0.027*
≥5 mg/mL	2.274 (1.163–4.448)	2.636 (1.118–6.217)
Cyfra 21-1		
<3.3 ng/mL	0.135	0.667
≥3.3 ng/mL	1.885 (0.821–4.328)	0.726 (0.169–3.120)
Tumor depth		
<10 mm	*0.030*	*0.052*
≥10 mm	2.207 (1.080–4.510)	2.560 (0.991–6.613)
Cyfra 21-1 and CRP	*0.019*	0.235
Cyfra 21-1 < 3.3 ng/mL, CRP < 5 mg/L	1	1
Cyfra 21-1 ≥ 3.3 ng/mL, CRP < 5 mg/L	0.575 (0.077–4.300)	0.000 (0.000)
Cyfra 21-1 < 3.3 ng/mL, CRP ≥ 5 mg/L	1.683 (0.761–3.722)	2.578 (1.036–6.414)
Cyfra 21-1 ≥ 3.3 ng/mL, CRP ≥ 5 mg/L	4.065 (1.610–10.261)	1.928 (0.426–8.726)

*ECS* extra-capsular spread

^a^T1–T2

^b^T3–T4

**Fig. 3:**
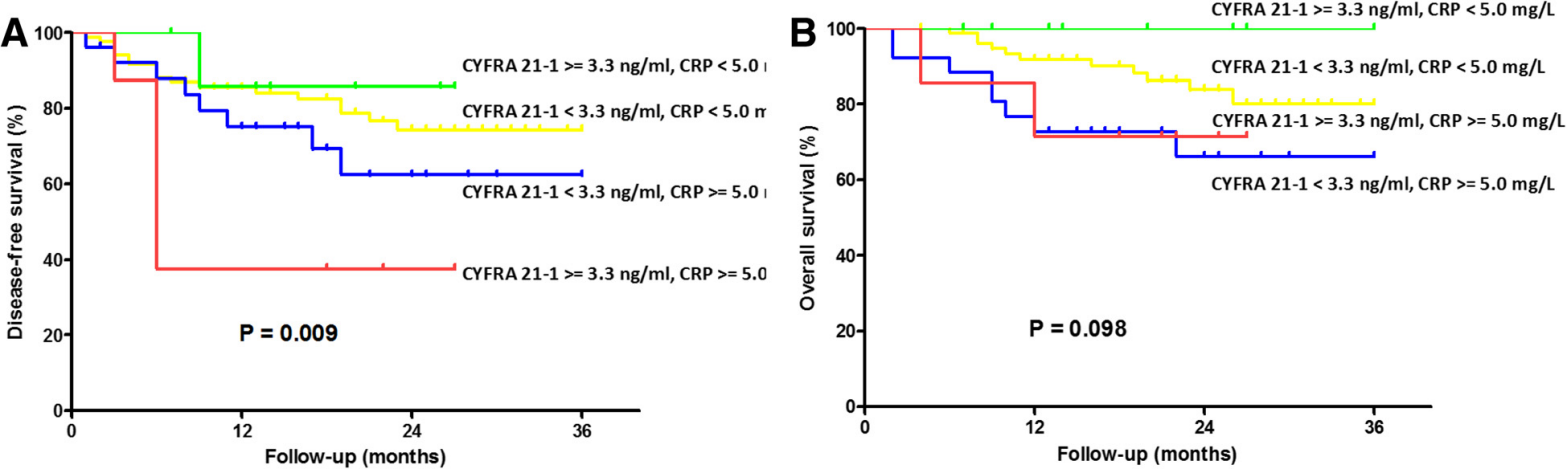
Survival curves in 130 OSCC patients related to the preoperative CRP and CYFRA 21-1 level. **a** The lower CRP and lower CYFRA 21-1 level group showed significantly better DFS compared to the higher CRP and higher CYFRA 21-1 level group (*P* = 0.009). **b** The lower CRP and lower CYFRA 21-1 level group showed significantly better OS compared to the higher CRP and higher CYFRA 21-1 level group (*P* = 0.098)

When the survival rates of the four groups were compared, the DFS of the higher CYFRA 21-1 and higher CRP level group was significantly lower than that of the other groups (log-rank test, *P* = 0.009, Fig. 3a). In addition, the OS was lower in the same group than that in the other groups (log-rank test, *P* = 0.098, Fig. 3b) but does not reach statistical significance (Table 4).

## Discussion

Deng et al. analyzed 142 HNSCC cases, and they found that the positive rates of CYFRA 21-1 increased with progression of HNSCC; serum CYFRA 21-1 levels were related to the primary tumor status and nodal status (*N*) (*P* < 0.001), but not related to patient age, gender, smoking and drinking habit, or histopathological grade (*P* > 0.05). The CYFRA 21-1 in HNSCC decreased significantly (*P* < 0.001) after treatment [30]. Doweck et al. reported that CYFRA 21-1 can be used in HNSCC at a sensitivity of 60 %, with a good correlation with tumor stage and an inverse correlation with the grade of tumor differentiation [14]. They suggested that CYFRA 21-1 was a good marker for HNSCC. In our study, CYFRA 21-1 was not associated with bone, skin, or perineural invasion. The only significant parameter is the lymph node metastasis. It implies that CYFRA 21-1 could be released into the bloodstream by metastatic tumor cells.

Regarding the role of CYFRA 21-1 in predicting prognosis, the results are positive in the literature [14, 32]. However, the prognostic role of CYFRA 21-1 in our study was statistically insignificant. There are two possible reasons: one is low sensitivity rate of elevated CYFRA 21-1. Eighteen patients (13.85 %) were found with elevated CYPFRA 21-1. The other is the cut-off values of CYFRA 21-1 in the literature are different. The results could be different when choosing different cut-off points. It is also apparent that there is contradictory information emerging from different laboratories. In Wang et al.’s review, different methods in the detection of CYFRA 21-1 including immunoradiometric assay, ECLIA, and enzyme-linked immunosorbent assay (ELISA) were used in the literature [33]. The use of CYFRA 21-1 in detecting OSCC could be limited because the increase in CYFRA 21-1 provides low sensitivity [18].

Serum CYFRA 21-1 level was linearly associated with CRP level (*P* = 0.010). As seen in the results, the elevated CRP was associated with bone invasion, skin invasion, and lymph node metastasis. CRP was elevated due to peri-tumor tissue destruction or lymph node metastasis. When we combined these two markers, a strong correlation was found between both higher CYPFRA 21-1 and CRP levels and tumor stage, nodal metastasis, skin invasion, and bone invasion (Table 3). In OSCC patients with lymph node metastasis, the serum CYPFRA 21-1 and CRP could be useful in stratifying the patients.

**Fig. 4:**
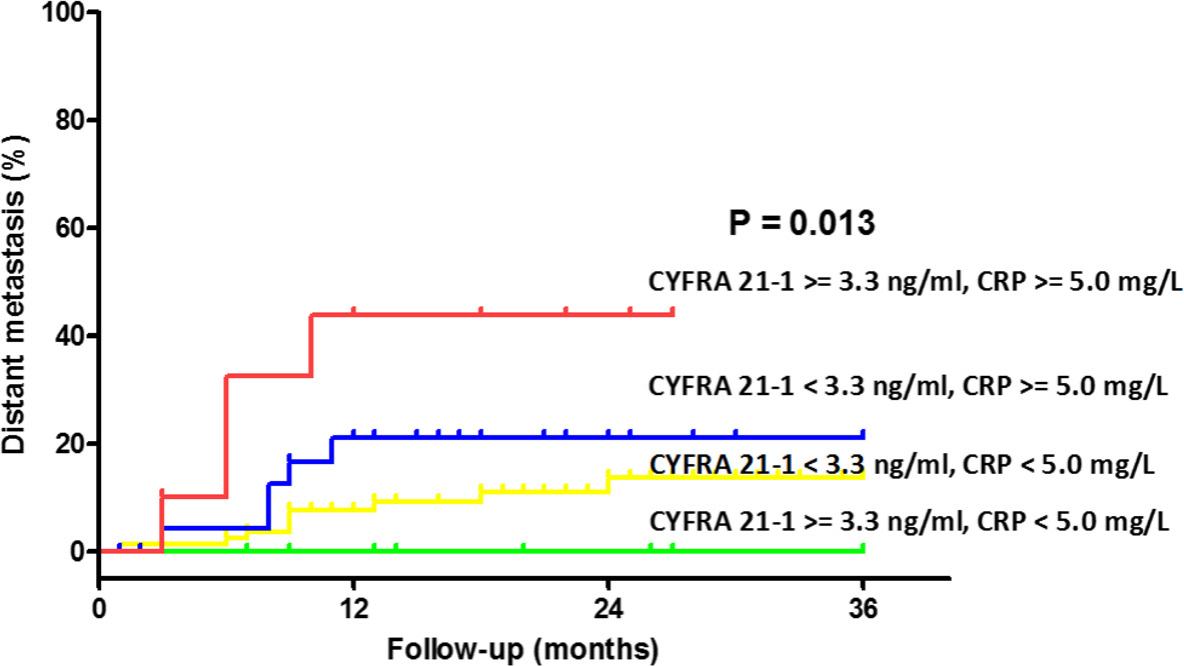
Distant metastatic rates in 130 OSCC patients related to the preoperative CRP and CYFRA 21-1 level. The higher CRP and higher CYFRA 21-1 level group showed significantly higher distant metastatic rate compared to the lower CRP or lower CYFRA 21-1 level group (*P* = 0.013)

Regarding patients’ survival, simultaneous elevation of the CYPFRA 21-1 and CRP level was related with worse DFS and OS in univariate analysis. Another prognostic end point is the interval between treatment and distant metastasis. We found that patients with higher CYPFRA 21-1 and CRP levels carry higher risks of distant metastasis (Fig. 4, log-rank test, *P* = 0.013, HR [95 % CI] 1.692 [1.097–2.414]). The elevation of CYPFRA 21-1 could be from the release of tumor antigen into the blood stream. Host immune system responds to tumor growth with elevated inflammatory cytokines (such as interleukin-6) and subsequently elevates the serum CRP levels [34]. The association of higher CYPFRA 21-1 and CRP with distant metastasis could be attributed to the advanced tumor stage or the existence of circulating tumor cells. Combination of these two markers provides clinicians clues of worse prognosis in OSCCs before surgeries.

We demonstrated that elevated CYFRA 21-1 levels in OSCC predicted nodal metastases in OSCC patients. The present study has further demonstrated the use of combined CYFRA 21-1 and CRP as a prognostic marker in OSCC and may be significant as a biomarker to predict prognosis and stratify patients for adjuvant therapies in the absence of traditional indications such as lymph node ECS. In this study, the follow-up period was relatively short and patient number was not large. In multivariate adjustment, the prognostic role of combined CYPFRA 21-1 and CRP in our analysis does not reach statistical significance. Our present study per se is preliminary. Further work and longer follow-up are required to elucidate the exact molecular mechanisms and clinical application of CYFRA 21-1 and CRP in OSCC.

## Conclusions

Preoperative CYFRA 21-1 serum concentration was related with lymph node metastasis. CRP level predicts greater extent of tumor destruction including bone invasion, skin invasion, tumor status, and lymph node metastasis. Combining the CYPFRA 21-1 and CRP levels predicts higher risks of disease recurrence and distant metastasis. Preoperative CYFRA 21-1 and CRP levels are thus probable candidates as biomarkers for risk stratification in OSCC.
